# Plant Functional Group Composition Modifies the Effects of Precipitation Change on Grassland Ecosystem Function

**DOI:** 10.1371/journal.pone.0057027

**Published:** 2013-02-20

**Authors:** Ellen L. Fry, Pete Manning, David G. P. Allen, Alex Hurst, Georg Everwand, Martin Rimmler, Sally A. Power

**Affiliations:** 1 Department of Life Sciences, Imperial College London, Ascot, Berkshire, United Kingdom; 2 Grantham Institute for Climate Change, Imperial College London, London, United Kingdom; 3 NERC (Natural Environment Research Council) Centre for Population Biology, Imperial College London, Ascot, United Kingdom; 4 Agroecology, University of Göttingen, Göttingen, Germany; 5 Department of Ecological Modelling, Bayreuth University, Bayreuth, Germany; 6 Hawkesbury Institute for the Environment, University of Western Sydney, Penrith, New South Wales, Australia; University of Alberta, Canada

## Abstract

Temperate grassland ecosystems face a future of precipitation change, which can alter community composition and ecosystem functions through reduced soil moisture and waterlogging. There is evidence that functionally diverse plant communities contain a wider range of water use and resource capture strategies, resulting in greater resistance of ecosystem function to precipitation change. To investigate this interaction between composition and precipitation change we performed a field experiment for three years in successional grassland in southern England. This consisted of two treatments. The first, precipitation change, simulated end of century predictions, and consisted of a summer drought phase alongside winter rainfall addition. The second, functional group identity, divided the plant community into three groups based on their functional traits- broadly described as perennials, caespitose grasses and annuals- and removed these groups in a factorial design. Ecosystem functions related to C, N and water cycling were measured regularly. Effects of functional groupidentity were apparent, with the dominant trend being that process rates were higher under control conditions where a range of perennial species were present. E.g. litter decomposition rates were significantly higher in plots containing several perennial species, the group with the highest average leaf N content. Process rates were also very strongly affected by the precipitation change treatmentwhen perennial plant species were dominant, but not where the community contained a high abundance of annual species and caespitose grasses. This contrasting response could be attributable to differing rooting patterns (shallower structures under annual plants, and deeper roots under perennials) and faster nutrient uptake in annuals compared to perennials. Our results indicate that precipitation change will have a smaller effect on key process rates in grasslandscontaining a range of perennial and annual species, and that maintaining the presence of key functional groups should be a crucial consideration in future grassland management.

## Introduction

Grasslands provide an important range of ecosystem services, including forage production and carbon storage [1], but are often managed for food production with little consideration for biodiversity conservation, resulting in widespread declines in their species richness [2]. They are also threatened by climate change, including changes in precipitation patterns. For example, in southern England summer rainfall is projected to decrease in volume but occur in more extreme downpours, with more severe interim droughts, accompanied by increased winter rainfall [3]. Because grasslands respond directly to the volume, frequency and duration of precipitation, such changes will affect their species composition, rates of nutrient and carbon cycling and water relations, and could see them shift from carbon sinks to sources [4],[5]. Additionally, extended periods of soil drying and wetting can be detrimental to soil microbial communities.In severe cases this may lead to cell lysis and nutrient exudation, followed by leaching and reduced soil fertility. All these changes mean that climate change may ultimately result in further diversity loss in grassland communities [6],[7].

In the last twenty years,experiments that explore the interaction between precipitation change and plant functional diversity loss have demonstrated that species richness is positively correlated with drought resistance and rates of important ecosystem processes such as respiration and soil nutrient availability [8]–[10], but the underlying causes of this relationship are not fully understood. Meanwhile, in other areas of ecological research there have been numerous attempts to discover which functional traitsdrive ecosystem functioning [11]–[15]. Currently the links between these two fields of enquiry are not explicit,but making them so may yield a greater understanding of how ecosystems respond to climate change. Some climate manipulation studies demonstrate long-term effects of climate change upon function, even after the removal of stresses (e.g. drought) [6],[16],[17], whilst in others, recovery is rapid and has few long term effects [7]. This variability in ecosystem resilience may be caused by differences in the functional trait distributions of the systems measured, with certain combinations of functional effects trait values (including rooting depth, relative growth rates and nutrient turnover rates) offering greater resistance and resilience to climate stresses [18],[19]. For example, ecosystems containing high plant functional trait diversity could exhibit smaller changes to ecosystem function in response to changes in rainfall patterns than less diverse assemblages. One reason for this is that trait diversity both above- and belowground is likely to offer a greater variety of plant resource and water capture strategies, and a greater diversity of rhizosphere niches for soil microbes [20],[21]. The probability of including traits that directly provide resistance or resilience to drought (e.g. traits conferring drought tolerance or rapid regeneration) is also increased in diverse communities [19],[22]. Furthermore, under altered abiotic conditions, hitherto subdominant species may increase in abundance and offer higher resilience to adverse conditions (the ‘insurance effect’) [23].

Differences in the response of species and functional groups to climate change are likely to ultimately lead to changes in ecosystem function. Dominant species (particularly perennial-dominated communities) are possibly more vulnerable to climate changes because their resources are allocated to maintaining competitive superiority over other species rather than to resisting environmental perturbations [23]–[25]. In contrast, annual species with their short life cycles, rapid growth and prolific reproductive output are potentially more resilientand able to recover from extreme weather events [15],[26]. These functional groups are also likely to differ in the way in which they influence ecosystem function, and so ecosystems in which they are rare or absent are likely to function differently under climate change. However, such relationships remain hypothetical.Therefore, we established an experiment that combined the manipulation of a precipitation regime (as opposed to a drought event) with a diversity manipulation based on functional groups with known functional traits. Most grassland climate change studies to date have focussed on primary productivity, so we addressed a knowledge gap by placing a greater emphasis on changes in plant species composition, gas fluxes and nutrient cycling [27]–[29].

Functional identity was manipulated by selectively removing functional groupings of plant species to create a gradient of functional diversity. By classifying plant species into functional types based upon effects traits, and removing the groups in factorial combination, we aimed to investigate how the presence of certain trait suites canmodify the response ofecosystem function to an altered precipitation regime.

## Methods

### Study site

The experiment, which is known as DIRECT -DIversity, Rainfall and Elemental Cycling in a Terrestrial ecosystem- is located in south east England, in Silwood Park, Berkshire, UK (0°35′W, 51°25′N). The site containsa lowland mesotrophic *Holcus mollis*-*Agrostis capillaris* grassland (EUNIS code E2, (European Nature Information System, http://eunis.eea.europa.eu)) on a loamy sand soil. There are no protected or threatened species present. It is surrounded by a rabbit-proof fence, although there is some roe deer (*Capreolus capreolus* L.) browsing and mole (*Talpa europaea* L.) activity. The climate is temperate: rainfall averaged 833 mm yr^−1^ between 2000 and 2010, and temperatures averaged 4.8°C in January and 17.2°C in July over the same period [30]. The field was ploughed in October 2007, which removed most standing biomass, and was left to regenerate naturally.

### Experimental design

The experiment began in June 2008, when the roofs of the precipitation change treatment were first raised. Measures were taken regularly, from October 2008 to September 2010. The experiment had a factorial, randomised block design consisting of two levels of rainfall (precipitation change rainfall and control) combined with seven combinations of three plant functional trait group (present/absent). The latter comprised every possible combination except none present (bare earth). This generated a diversity gradient of 1–3 groups. Four blocks, each containing one replicate of each treatment combination, were arranged in a row from east to west, resulting in 56 plots. This blocking accounted for a shallow incline across the site (Figure S1). Each plot was 2.4 m×2.4 m, with a 70 cm buffer zone to account for lateral drift of rain; ecosystem function measures were taken in a central 1 m×1 m central area within each plot.

### Precipitation change treatment

The rainfall treatment was based upon end of the century predictions from climate models using A2 scenarios from the IPCC 4^th^ Assessment Report [31]; these project that by 2080–2099 south-east England will experience a reduction of ∼30% rainfall volume during the summer months (June, July, August; JJA) relative to the 1961–1990 baseline.These rainfall events are also likely to become less frequent, and concentrated into more intense downpours [3]. All 56 plots were covered with a rain shelter from June 1^st^ to August 31^st^ each year (Figure S2). The shelters were open sided, and covered with transparent corrugated Corolux PVC, 0.8 mm thick. All the rain was removed from the precipitation change plotsand collected in individual water butts. In the control plots, roofs had approximately 100 2.5 cm diameter holes to allow rainwater to pass through. In the precipitation change treatment, if less than 20 mm fell in 24 hours, 50% of the water was reapplied manually and the rest discarded. If more than 20 mm fell, the full amount was reapplied. Based on historic rainfall data for the site, this was estimated to approximate to a net reduction of 30% volume over the growing season. Projections for the winter (Dec, Jan, Feb; DJF) consist of a 10–15% volume increase for southern England, with frequency and intensity remaining approximately the same as at present. DJF rainfall treatments were applied by collecting control rainfall in weather-resistant water trays placed adjacent to each precipitation change treatment plot, with surface area of 15% of plot size (approx. 8640 cm^2^). The water collected was reapplied to all precipitation change treatment plots after every rainfall event from December 1^st^ to February 28^th^ each year. The PVC roofs led to an overall reduction of 34% photosynthetically active radiation (PAR) in all plots, but this was the same in both treatments as the holes had little effect upon light transmission (Analysis of variance of comparable readings under control and precipitation change roofs F_1,53_ = 0.79, p = 0.377). There was a <1% increase in temperature under the shelters compared with outside but humidity was unaffected.

### Functional group identity treatment

For the functional group identity treatment three plant trait groups were derived using a divisive hierarchical cluster analysis based on functional effects trait data. These data were obtained by growing all common grassland species from the local species pool to maturity in a greenhouse and measuring above and belowground biomass (AGB and BGB respectively), leaf nitrogen (N) content (LNC), specific plant area (plant area/AGB) (SPA), photosynthetic rate (*A*) and evapotranspiration rate. Additional trait data on plant lifespan and N fixing capacity were obtained from the USDA plants database [32]. Relationships between greenhouse and field traits have been the subject of some contention, but there is now compelling evidence that the two are closely related [33]. Accordingly, greenhouse-derived trait measures can be used as a measure of relative differences between species trait values in field conditions with reasonable confidence. The cluster analysis was set to divide the species into three groups (Table S1). The first of these comprised perennial grasses, forbs and legumes (hereafter FG1), whose distinguishing traits included higher SPA, LNC and a more perennial growth habit than the other groups (Table 1), characteristics which are expected to result in faster ecosystem process rates and higher and more continuous net turnover of plant material [21],[34],[35]. The second group (FG2) consisted of caespitose grasses and two forbs, with very high AGB and BGB, and low LNC. Presence of this group is expected to result in large amounts of poor quality litter inputs to the soil. The third group (FG3) consisted of annual forbs, grasses and legumes, with low SPA and biomass but high LNC. Presence of these traits, coupled with their short lifespan, could potentially result in tolerance of environmental stress as well as rapid growth and recovery from drought. Strong seasonal trends in function were expected where FG3 is present with senesced material decomposing rapidly in autumn and high nutrient and CO_2_ flux rates in the spring and early summer when germination and growth occur.

**Table 1:** Trait means for each functional group.

Trait	FG1	FG2	FG3
Plant height (cm)	34.3±3.8	65.9±13.3	51.2±5.4
Root depth (cm)*	100+	Variable	0–10
Aboveground biomass (g)	1.9±0.2	9.0±1.6	2.8±0.3
Belowground biomass (g)	1.9±0.5	5.0±1.0	1.0±0.20
Specific plant area (mm^2^ mg^−1^)	17.4±1.3	13.8±3.4	11.9±1.1
Leaf N content (mg kg^−1^)	2359±229	1357±152	2203±160
Leaf N∶P ratio	8.1±3.0	4.9±0.6	7.8±0.6

Trait means ± standard error for the functional groups from plants grown in a greenhouse on mesotrophic acid soil. FG1 is dominated by perennial forbs and grasses, FG2 is dominated by caespitose grasses, while FG3 has annual grasses, forbs and legumes.

* information taken from the Ecoflora database (http://www.ecoflora.co.uk/).

The three groups were combined into every possible combination(except for the absence of all) - three individual groups, three combinations of two, one combination of three. Plant functional group identity treatments were implemented by weeding out unwanted species. All plots also contained the dominant of the site *Holcus mollis* (FG1), as its removal would have caused such significant disturbance that the functional group identity treatment would be highly confounded with this. In the absence of *H. mollis* effects of FG1 removal may have been stronger but there is no evidence that FG1 possesses any unique functional traits and therefore the removal of all except one FG1 species should be viewed as an alteration of the distribution of traits within the community. Weeding took place throughout the experiment, with major efforts in August 2008, June 2009 and May 2010. Vegetation was surveyed before the initial weeding effort, and non-*Holcus mollis* cover was comprised of 87% FG1, 5% FG2 and 8% FG3. Biomass removal was initially large (up to 13.2 kg per plot where FG1 was removed in August 2008) but declined substantially throughout the experiment as adult plants were no longer present. Subsequent weeding efforts only required the removal of invading seedlings so did not appreciably affect total cover, which had recovered by May 2009 in all functional group treatments (Figure S3). Post-weeding total cover in September 2010 was 76% when FG1 was present and 69% when it was absent (F_1,42_ = 4.29, p = 0.05, see below for statistical methods, and Table S2 and Figure S3 for more complete cover data).

### Field measures

Rainfall data for the duration of the experiment were obtained from an onsite Vantage Pro wireless weather station (Davis Instruments, USA) and daily measures were taken from a rain gauge to determine the amount of rainfall to be applied to treatment plots. The average soil moisture content of each plot to 10 cm depth was measured weekly using a ThetaProbe Soil Moisture Meter HH2 with ML2x probe (Delta-T, UK) at a distance of 1 m from the plot edge on all four sides.

Vegetation surveys were carried out in October 2008, May 2009, September 2009, May 2010, July 2010 and September 2010. Visual estimates of percentage cover of each species were taken from the central 1 m^2^ of each plot in order to determine whether there was an effect of the treatments (both precipitation and functional group removals) upon species richness, individual species abundance and total vegetation cover (Figure S3). The cover of bare ground and dead plant material was also recorded.Total vegetation cover was derived from the sum of individual species cover estimates, and was used as a proxy, non-destructive measure of aboveground biomass.

Decomposition rate measurements began in December 2008. Two grams of dried (80°C for 24 h), cut leaf samples of *Holcus mollis* were placed in 8 cm×8 cm mesh bags (1 mm mesh size, Normesh, Oldham, UK) and secured to the soil surface in each plot. Three bags were placed in each plot and one was removed in each of March, June and September 2009. On collection, new biomass growing through the mesh was removed and the remaining material was dried at 80°C for 24 hours and weighed to determine relative mass loss.

CO_2_ and water flux rates were measured using a transparent Perspex chamber (area 300 cm^2^, volume 9000 cm^3^) attached to a CIRAS-1 infra-red gas analyser (IRGA), (PP Systems, Hitchin, UK), which was clipped onto PVC ring collars inserted into the soil to a depth of 5 cm (20 cm diameter, 10 cm long) to create a sealed area over the plants and soil. In light conditions, the returned values were net ecosystem CO_2_ exchange (NEE), (mg CO_2_ m^−2^ s^−1^). This was repeated with an opaque cover to obtain an estimate of dark ecosystem respiration (R_eco_). To obtain ecosystem photosynthetic rate (*A*), NEE was subtracted from R_eco_. These measures were taken monthly during the summerand in alternate months through the winter, between March 2009 and September 2010. Soil moisture, PAR; (Skye Instruments, Wales) and soil temperature (Hanna, Bedfordshire, UK) were measured concurrently as covariates.

Extractable soil ammonium (NH_4_^+^), nitrate/nitrite (NO_3_^−^/NO_2_^−^) and phosphate (PO_4_^3−^) concentrations of fresh soil were determined in December 2008 and 2009, March 2009 and 2010, and monthly from May–September in 2009 and 2010. Soil samples were taken (0–5 cm depth) from five separate areas in each plot and mixed to create a composite sample. Soils were then extracted using 1 M potassium chloride (75 ml KCl: 20 g fresh soil) solution for NH_4_^+^ and NO_3_^−^/NO_2_^−^ and Truog's solution for PO_4_^3−^ (150 ml Truog's solution: 10 g soil), [36], and analysed colourimetrically using a Skalar SAN^++^ auto-analyser (Skalar, York, UK). Precision was verified by repeating 5% of the samples as analytical replicates and including one matrix blank per 20 samples. Soil moisture was determined for each sample in order to express values as mg kg^−1^ dry weight. NO_2_^−^ concentrations were negligible so oxidised N will be referred to as NO_3_^−^ hereafter.

### Statistical analysis

The effect of the rainfall treatment on light interception by the shelters (see above) and soil moisture content was tested by one-way analysis of variance (ANOVA) using R2.12.0 [37] on averaged plot level data at each time point, with block as an error term [38]. Vegetation cover was analysed in order to examine the effect of the treatmentsover time. We used a linear mixed effects model (LME) across all time points. Block (four levels) and plot were included asrandom effects, and the main effects of sampling month, precipitation change treatment (two levels) and a binary presence/absence term for each functional group were calculated alongside two and three-way interaction terms between the treatments. The models excluded three-way interactions between all three functional groups, which were not possible to estimate due to the design of our experiment. This technique was also used to evaluate treatment effects upon species richness, and all individual species found in the plots.

Decomposition rates (arcsine transformed percentage mass loss) were analysed using a LME model with main effects of precipitation, presence/absence of each functional group and first order interactions between all main effects, and block as a random term at each timepoint measured.We did not analyse these data with repeated measures methods (see above) as litter bags were all installed at the same time and sub-sets harvested sequentially.

Repeated measures LMEs were carried out to test whether there was an effect of rainfall regime and functional group identity upon ecosystem functions (photosynthetic rate, ecosystem respiration, evapotranspiration, and extractable N and P) over the course of the whole study. These models included block and plot as random effects. An ANOVA was performed on each of the LME models. Following the repeated measures analysis, each time point was evaluated separately using LME so that the timing of significant effects could be evaluated.These models were identical in structure to those for vegetation cover.

## Results

### Soil moisture content

**Figure 1:**
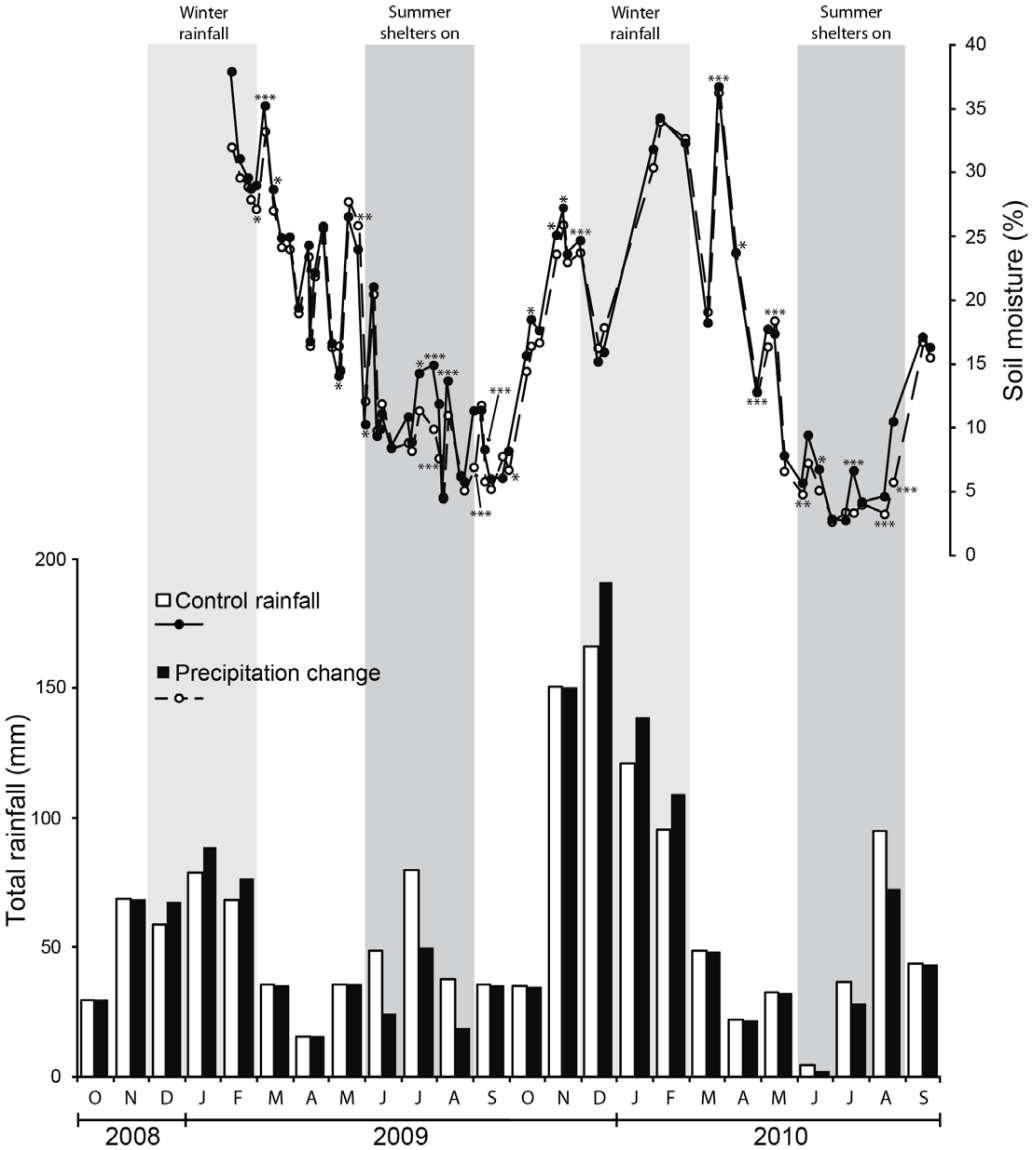
Rainfall applied to treatments during the measurement period of the experiment. Significant differences in soil moisture between precipitation treatments are represented by asterisks * = p<0.05, ** = p<0.01, *** = p<0.001.

The winter and summer phases of the precipitation change treatment had clear measureable effects on soil moisture (Figure 1). In the winter these effects were manifested with a time delay of around 60 days from the change in rainfall pattern. In the first winter of the experiment (2008–2009) the precipitation change treatment plots received 15% more rain than the control plots (Table 2). This resulted in significantly wetter soils in February of 2009, though in general effects were small throughout the rest of the period. In the second summer (2009), precipitation change treatment plots received 38.1% less rainfall than control plots and had significantly lower soil moisture levels all the way through until November, when high natural rainfall volumes raised soil moisture contents in both treatments. The second winter (2009–2010) was exceptionally cold and wet (Figure 1, Table 2). There was a clear lag-time between the high rainfall in December and a corresponding change in soil moisture, with a peak seen in February. After this, the soils dried rapidly, though carry-over effects of the winterprecipitation change treatment were still evident throughout the spring of 2010. The third summer (2010) was relatively dry but had three heavy rain events rather than two, so a higher proportion of total rainfall was applied to the precipitation change treatment plots (75% of 136 mm = 103 mm). As with 2009, the summer of 2010 showed highly significant treatment effects on soil moisture, with the precipitation change treatment being much drier throughout the summer. Unlike in the winter periods, a lagged effect on moisture was not apparent. No significant effects of functional group treatments were apparent for soil moisture throughout the experimental period.

**Table 2:** Total seasonal rainfall inputs throughout the experiment.

Year	Season	Precipitation change rainfall volume (mm)	Control rainfall volume (mm)
2008	Summer	128	222
2008–9	Winter	233	206
2009	Summer	93	166
2009–10	Winter	440	382
2010	Summer	103	136

### Plant community composition

The precipitation change treatment had significantly lower vegetation cover than the control throughout the experiment (Table 3). Plots where FG1 species were removed had significantly lower vegetation cover, although this appears to be mainly caused by a large difference in October 2008 following the first weeding occasion (Table 3); after this the difference in cover was small compared with other FG treatments (Figure S3). Note that when FG1 is described as absent or removed, this does not include the dominant at the site *H. mollis*, which belonged to FG1 but was allowed to remain in all plots. Later in the experiment significant effects of FG1presence on cover were observed in the May vegetation surveys but these were likely to be due to overwintering of perennials and dieback of annuals because weeding efforts were very small (Table S2). FG2 and FG3 removal did not significantly lower total plot cover.

**Table 3:** Results of linear mixed effects models testing precipitation change (PC) and functional group (FG) treatment effects upon vegetation cover and species richness.

Treatment		Vegetation cover		Species richness	
	d.f	F	p	F	P
*Intercept*	1	**889.05**	**<0.001**	**258.50**	**<0.001**
PC	1	**94.88**	**<0.001**	0.12	0.728
FG1 present	1	**32.54**	**<0.001**	0.00	0.990
FG2 present	1	0.01	0.925	0.35	0.556
FG3 present	1	0.14	0.713	1.99	0.166
PC x FG1	1	0.81	0.372	1.91	0.175
PC x FG2	1	0.04	0.848	0.29	0.596
PC x FG3	1	0.85	0.362	1.54	0.222
FG1 x FG2	1	**5.22**	**0.028**	0.14	0.710
FG1 x FG3	1	0.22	0.642	1.57	0.218
FG2 x FG3	1	0.90	0.348	0.01	0.926
Residuals	42				
Month	5	**47.48**	**<0.001**	2.08	0.069
FG1 x Month	5	**16.39**	**<0.001**	1.72	0.130
FG2 x Month	5	**2.49**	**0.033**	0.62	0.683
FG3 x Month	5	0.38	0.862	1.35	0.245
PC x Month	5	30.01	**<0.001**	0.90	0.481
PC x FG1 x Month	5	1.47	0.202	1.26	0.281
PC x FG2 x Month	5	0.49	0.781	0.47	0.795
PC x FG3 x Month	5	0.77	0.574	1.62	0.157
FG1 x FG2 x Month	5	2.21	0.055	0.22	0.953
FG1 x FG3 x Month	5	1.13	0.345	**2.38**	**0.040**
FG2 x FG3 x Month	5	0.75	0.586	0.01	1.000
Residuals	225				

Species richness was not significantly affected by the treatments, nor did it change over time (Table 3), averaging seven species per m^2^ throughout. *Holcus mollis*, while initially relatively abundant in all plots, averaging 45.5% cover, declined consistently through the experiment, and by September 2010 averaged 12% cover (Table S3, Figure S4).

When each species was tested individually for sensitivity to precipitation change over time, only one out of the 52 species recorded at the site over the duration of the experiment was significantly affected. *Rumex acetosella*, while always having very low cover, was almost completely lost in precipitation change treatment plots (0.12±0.04% cover compared with 0.63±0.12% in control plots, F_1,51_ = 8.53 p = 0.005).

### Decomposition rates

**Figure 2:**
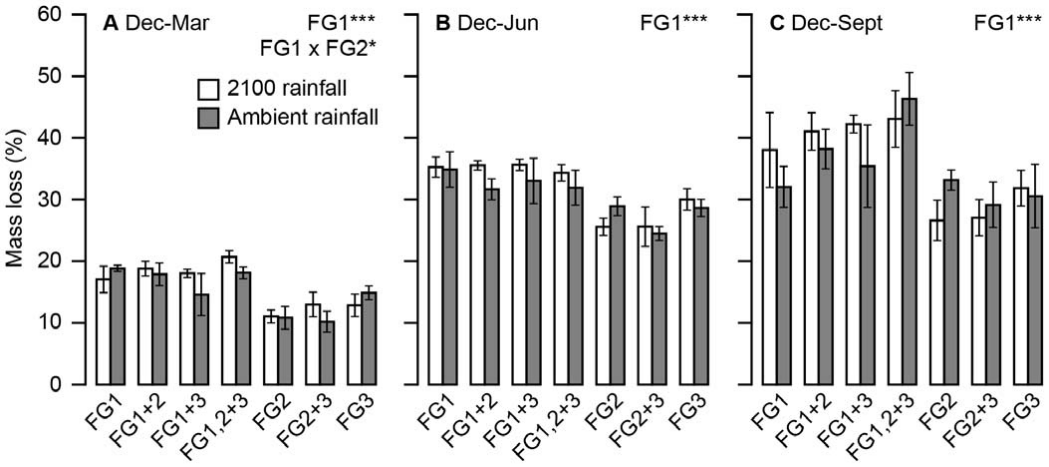
Effect of precipitation change and functional identity on decomposition of *Holcus mollis* at different time points. Decomposition of *H. mollis* litter in 2009 for all fourteen treatments. a) Dec–Mar F_1,49_ = 32.87, p<0.001, b) Dec–Jun F_1,49_ = 40.23, p<0.001, c) Dec–Sept F_1,49_ = 20.31, p<0.001. Error bars represent ±1 SEM.

Functional group identity was a significant driver of litter mass loss, although there was no significant effect of precipitation change. Decomposition rates were consistently higher when FG1 species were present throughout the nine months of measurement (Dec–Mar = 29.1%, Dec–Jun = 14.6%, Dec–Sept = 19.6% higher when present, Table 4; Figure 2a–c). This group is characterised by short, N-rich species with deep roots (Table 1). However, there was an interaction between the presence of FG1 and FG2 in the Dec–Mar period (Table 4; Figure 2a). When FG2 was absent, and FG1 was present, decomposition was close to the average in this period.When FG2 was present in the plots but FG1 was absent, decomposition was slow, possibly as a result of the production of large amounts of low quality litter; FG2's distinguishing traits include a very low LNC and high shoot and root biomass (Table 1). This effect was overwhelmed by the more abundant FG1 where it was present; when FG1 and FG2 were both present, decomposition was high.

**Table 4:** Summary of treatment effects upon decomposition from mixed effects models.

Date	Treatment	d.f.	F	p
Dec–Mar 09	*Intercept*	1	**2948.92**	**<0.001**
	PC	1	0.92	0.342
	FG1	1	**33.93**	**<0.001**
	FG2	1	0.00	0.963
	FG3	1	0.25	0.623
	PC x FG1	1	0.21	0.647
	PC x FG2	1	0.87	0.358
	PC x FG3	1	1.59	0.214
	FG1 x FG2	1	**4.69**	**0.036**
	FG1 x FG3	1	0.23	0.638
	FG2 x FG3	1	1.17	0.287
	Residuals	42		
Dec–Jun 09	*Intercept*	1	**4194.75**	**<0.001**
	PC	1	1.21	0.278
	FG1	1	**40.05**	**<0.001**
	FG2	1	3.62	0.064
	FG3	1	0.61	0.440
	PC x FG1	1	1.55	0.220
	PC x FG2	1	0.00	0.960
	PC x FG3	1	0.95	0.335
	FG1 x FG2	1	1.31	0.260
	FG1 x FG3	1	0.50	0.486
	FG2 x FG3	1	0.01	0.921
	Residuals	42		
Dec–Sept 09	*Intercept*	1	**1641.20**	**<0.001**
	PC	1	0.13	0.721
	FG1	1	**20.94**	**<0.001**
	FG2	1	1.11	0.298
	FG3	1	1.79	0.188
	PC x FG1	1	1.74	0.194
	PC x FG2	1	2.26	0.140
	PC x FG3	1	0.00	0.989
	FG1 x FG2	1	1.95	0.170
	FG1 x FG3	1	1.59	0.215
	FG2 x FG3	1	0.05	0.820
	Residuals	42		

FGx refers to the presence of the functional group in question, PC to the precipitation change treatment.

### Ecosystem CO_2_and water fluxes

**Figure 3:**
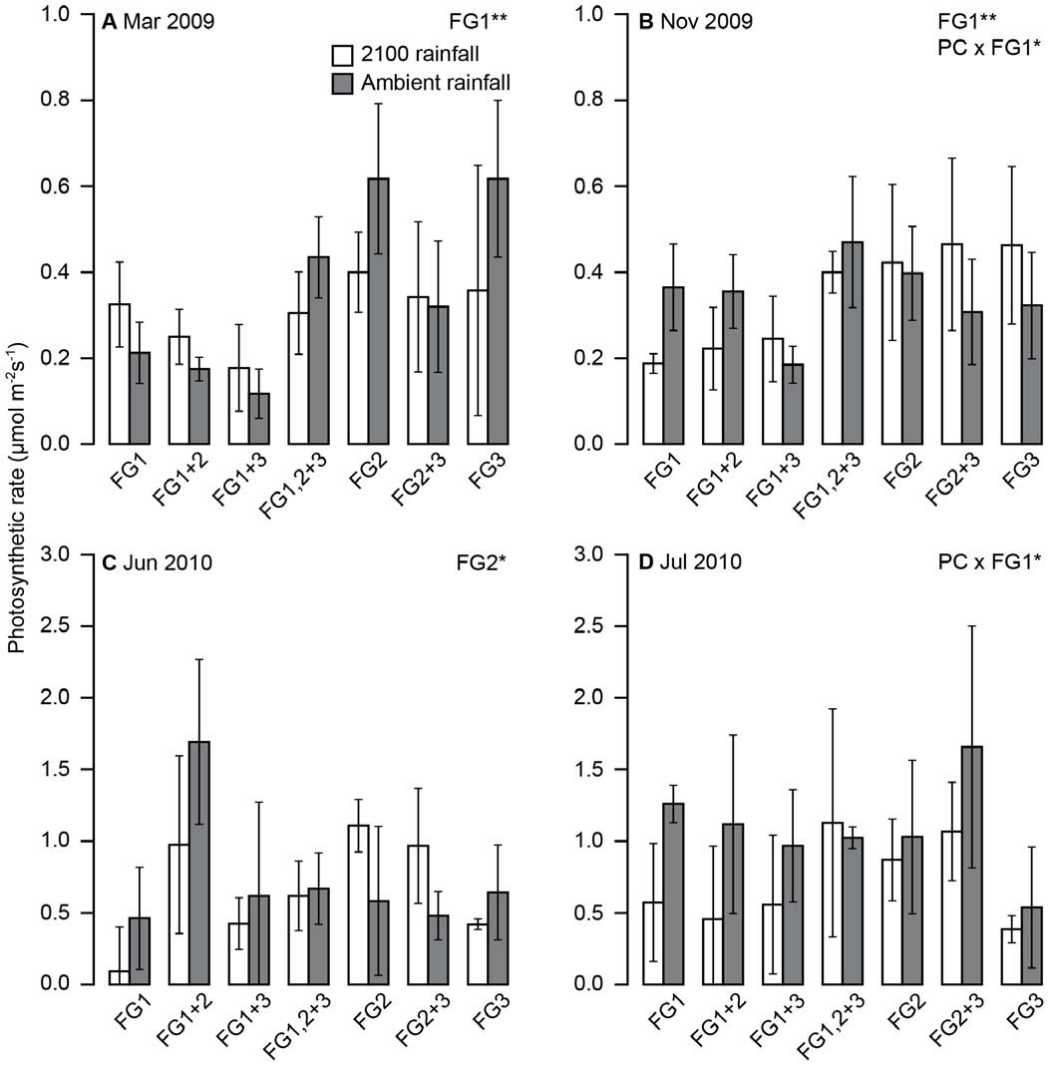
Effect of precipitation change and functional identity on photosynthetic rate at different time points. The response of photosynthetic rate to precipitation change (PC) and functional group (FG) identityat four time points through the experiment. a) March 2009 (presence/absence of FG1, F_1,42_ = 9.152, p = 0.004), b) November 2009 (interaction between PC and FG1, F_1,42_ = 4.831, p = 0.033), c) June 2010 (presence/absence of FG2, F_1,42_ = 4.610, p = 0.037), d) July 2010 (interaction between PC and FG1, F_1,42_ = 5.552, p = 0.004). Error bars represent ±1 SEM.

Changes to C fluxes caused by precipitation change were strongly modified by plant functional group identity. The responses of R_eco_ and *A* were very similar, indicating that both fluxes were driven primarily by plant community activity. Under ambient conditions, significantly higher flux rates were associated with the presence of FG1. Across the experiment we observed average photosynthetic rates of 1.04±0.08 mg CO_2_ m^−2^ s^−1^ when FG1 was present compared with 0.89±0.07 mg CO_2_ m^−2^ s^−1^ in its absence (Table 4). The main significant effects occurred in the first year after the winter and summer treatments ceased (March and November 2009, Figure 3a–b), and during the summer rainfall treatment in the second year (June and July 2010, Figure 3c–d). For the most part, plots containing FG1significantly differed in their photosynthesis to those without it, and there was a significant precipitation change effect in November 2009 and July 2010. In March 2009, photosynthetic rate was lower in plots where perennial species were present than in plots where there were mainly germinating annuals and caespitose grasses (Figure 3a). This was also the case in November, after the summer drought treatment had ended, and this was associated with a precipitation change effect. Plots with perennial FG1 species that had been exposed to the 2100 treatment had lower overall photosynthetic rate (Figure 3b). In June 2010, a particularly dry month at the beginning of the summer precipitation treatment (Figure 1), effects of FG1 had been superseded by FG2 (Figure 3c).The average photosynthetic rate was higher when FG2 species were present in the plots, although this appeared to be largely driven by very high photosynthetic output in plots where FG1 and FG2 were present together. However, in July 2010 the interaction between FG1 and precipitation change had returned, following a similar pattern to November 2009 (Figure 3d). The different groups had different root allocations (Table 1), which indicate that most root biomass for FG1 was distributed deeper in the soil than the other groups, and was likely to result in less optimal use of small rainfall inputs.

**Figure 4:**
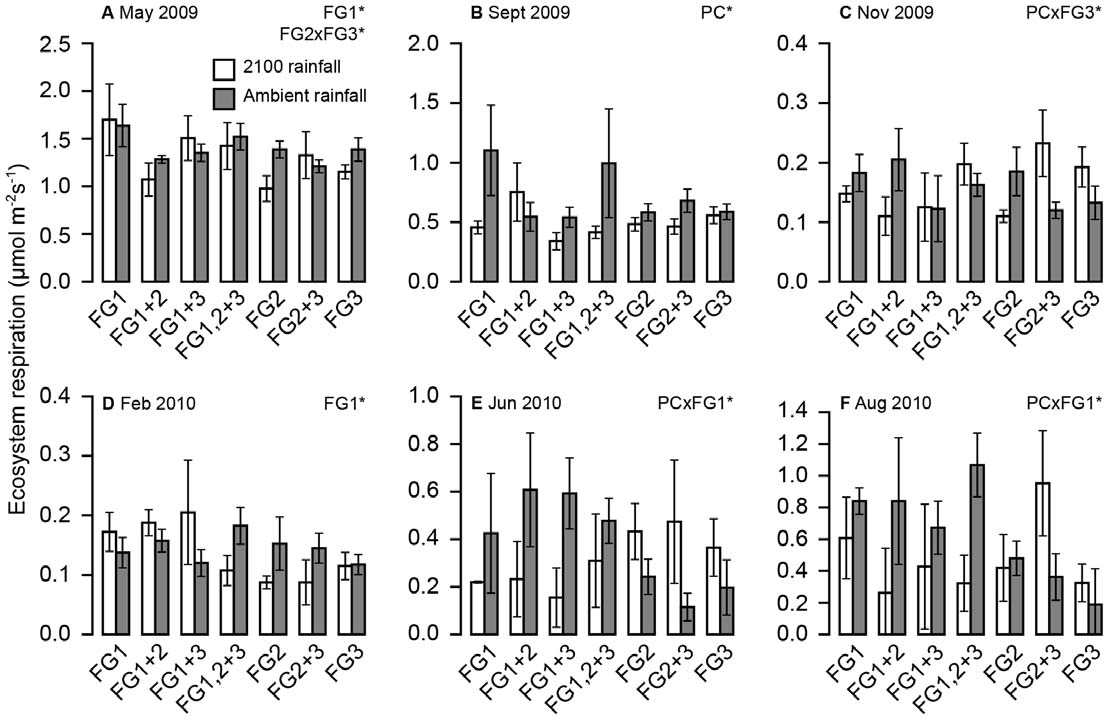
Effect of precipitation change and functional identity on ecosystem respiration at different time points. The response of ecosystem respiration to precipitation change (PC) and functional group (FG) identity at six time points through the experiment. a) May 2009 (presence/absence of FG1 F_1,42_ = 4.72, p = 0.036, interaction between FG2 and FG3, F_1,42_ = 5.031, p = 0.03), b) September 2009 (PC F_1,42_ = 4.596, p = 0.038), c) November 2009, (interaction between PC and FG3 F_1,42_ = 7.165, p = 0.010), d) February 2010 (FG1, F_1,45_ = 4.521_,_ p = 0.039), e) June 2010 (interaction between PC and FG1, F_1,45_ = 5.80, p = 0.020) f) August 2010 (interaction between PC and FG1 F_1,45_ = 6.46, p = 0.015).

R_eco_ averaged 0.61±0.05 mg CO_2_ m^−2^ s^−1^ when FG1 was present, and 0.46±0.04 mg CO_2_ m^−2^ s^−1^ when absent. This pattern was reversed under the precipitation change treatment, resulting in significantly lower rates of both fluxes when FG1 was present (0.7±0.09 mg CO_2_ m^−2^ s^−1^ (*A*), 0.41±0.05 mg CO_2_ m^−2^ s^−1^(R_eco_) under the precipitation change treatment, compared to values of 1.04±0.08 mg CO_2_ m^−2^ s^−1^ and 0.61±0.05 mg CO_2_ m^−2^ s^−1^for *A* and R_eco_, respectively, in control plots).The dominant trends showed that FG1 was associated with higher levels of R_eco_ than the other two groups, although there was often a significant effect of the precipitation change treatment, leading to reduced R_eco_, particularly through the summer months (Figure 4a,d–f). The precipitation change treatment was not a very strong driver on its own, and often only showed its effects in the presence or absence of certain functional groups.

Evapotranspiration rates were not strongly affected by functional group identity, but were significantly lower in the precipitation change treatment throughout the experiment (Table 5).

**Table 5:** Results of linear mixed effects models, testing precipitation change (PC) and functional group(FG) treatment effects upon carbon and water fluxes.

Treatment		Photosynthetic rate (*A*)		Ecosystem respiration (R_eco_)		Evapotranspiration (ET)	
	d.f.	F	p	F	p	F	P
*Intercept*	1	**875.59**	**<0.001**	**1016.98**	**<0.001**	**314.19**	**<0.001**
PC	1	**6.88**	**0.012**	**7.82**	**0.008**	**4.82**	**0.034**
FG1 present	1	0.56	0.458	3.83	0.057	0.19	0.669
FG2 present	1	0.65	0.425	0.15	0.700	0.02	0.881
FG3 present	1	0.16	0.688	0.73	0.398	0.83	0.367
PC x FG1	1	**7.21**	**0.010**	**12.02**	**0.001**	0.67	0.417
PC x FG2	1	0.11	0.742	0.17	0.679	0.51	0.478
PC x FG3	1	2.25	0.141	0.27	0.607	1.87	0.179
FG1 x FG2	1	2.27	0.139	0.32	0.575	1.05	0.311
FG1 x FG3	1	0.37	0.549	0.19	0.664	0.01	0.936
FG2 x FG3	1	0.04	0.843	2.87	0.098	2.10	0.155
Residuals	42						
Month	11	**42.19**	**<0.001**	**49.60**	**<0.001**	**21.85**	**<0.001**
FG1 x Month	11	1.01	0.439	0.50	0.901	0.48	0.917
FG2 x Month	11	**2.10**	**0.019**	0.77	0.675	1.59	0.097
FG3 x Month	11	0.52	0.893	0.23	0.995	0.57	0.858
PC x Month	11	0.73	0.712	0.71	0.728	0.70	0.735
PC x FG1 x Month	11	1.19	0.294	1.64	0.085	1.24	0.260
PC x FG2 x Month	11	0.28	0.990	0.66	0.780	0.82	0.624
PC x FG3 x Month	11	0.64	0.798	0.57	0.855	0.81	0.634
FG1 x FG2 x Month	11	0.91	0.534	0.60	0.830	0.70	0.742
FG1 x FG3 x Month	11	0.58	0.846	0.97	0.472	0.75	0.688
FG2 x FG3 x Month	11	0.61	0.821	0.97	0.475	0.93	0.509
Residuals	495						

### Extractable nutrient concentrations

**Figure 5:**
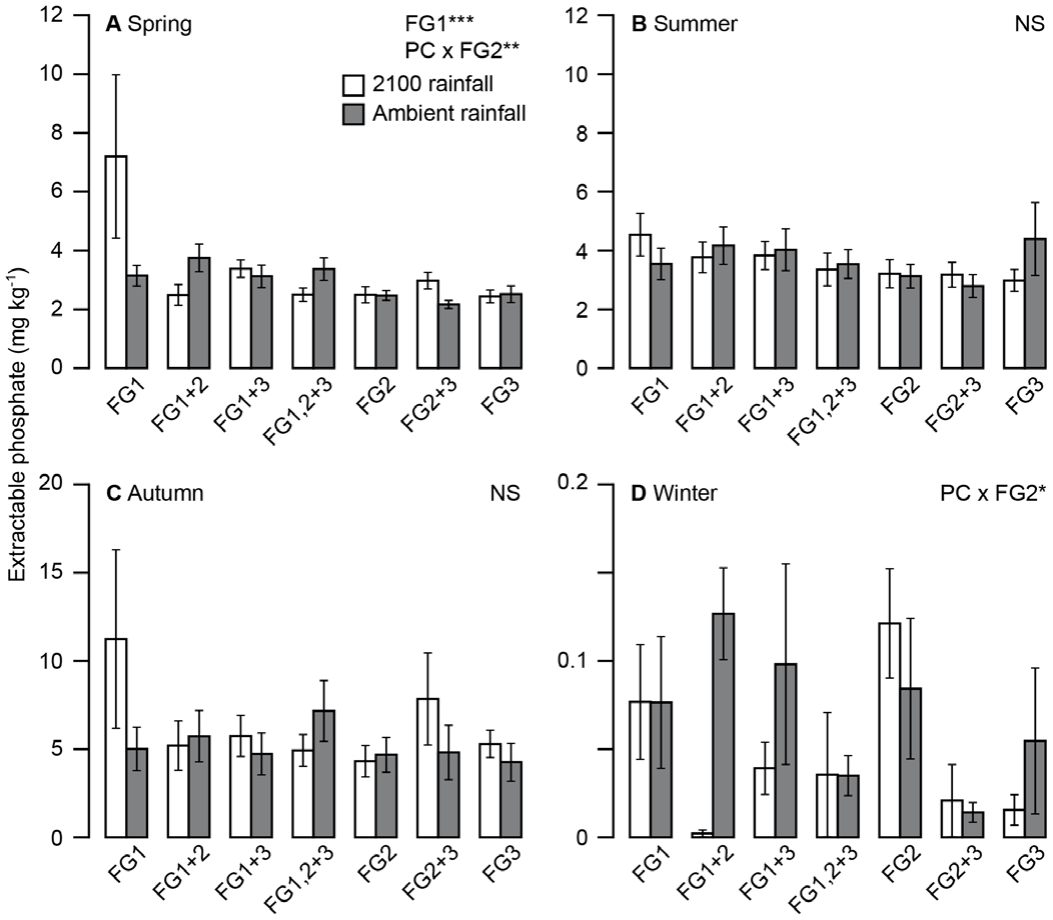
Effect of precipitation change and functional identity on extractable soil P in different seasons. The response of soil extractable P to precipitation change (PC) and functional group (FG) identity in each season. a) Spring (presence/absence of FG1, F_1,154_ = 14.1, p = 0.0003, interaction between PC and FG2, F_1,45_ = 6.88, p = 0.0096), b) Summer (NS), c) Autumn (NS), d) Winter (interaction between PC and FG2 F_1,45_ = 5.005, p = 0.0303).

Soil extractable NH_4_^+^ levels were not significantly altered by precipitation change or functional group identity (Table 6) throughout the experiment. Extractable NO_3_^−^ was affected by an interaction between precipitation change and FG1; NO_3_^−^ was slightly lower in plots where FG1 was absent. In contrast, extractable P concentrations were very strongly affected by precipitation change and presence of various functional groups across the seasons (Table 6; Figure 5a–d). Concentrations of extractable P were generally low throughout the experiment, with trace (<0.01 mg kg^−1^) amounts in the soil in February 2009, increasing to 43.1 mg kg^−1^ in September 2010, although average P concentrations for the experimentwere low, at 3.56 mg kg^−1^.There was a highly significant interaction between FG2 presence and precipitation change. During spring, if FG2 was absent there was a higher concentration of P in the soil of precipitation change plots (i.e. those which had received higher winter rainfall), (Figure 5a). In the summer and autumn months P availability was not significantly affected by the treatments (Figure 5b,c). In the winter, soil P availability was once again affected by the nutrient-poor FG2 species and an interaction with the precipitation change treatment (Figure 5d). The wetter 2100 treatment was associated with almost total loss of P from the system when FG2 was present, although higher concentrations were found in ambient plots. Overall, FG2 presence and the precipitation change treatment appeared to affect P concentrations during the wetter months, and have no effect during the warmer summers.

**Table 6:** Results of linear mixed effects models testing precipitation change (PC) and functional group (FG) treatment effects uponseasonal soil extractable nutrient concentrations.

Treatment		Extractable NH_4_^+^		Extractable NO_3_^−^		Extractable PO_4_^−^	
	d.f.	F	p	F	p	F	p
*Intercept*	1	**1305.27**	**<0.001**	**876.46**	**<0.001**	0.08	0.774
PC	1	0.02	0.901	1.56	0.218	1.18	0.284
FG1 present	1	1.65	0.206	0.89	0.351	1.89	0.177
FG2 present	1	1.28	0.265	0.00	0.952	1.17	0.286
FG3 present	1	0.00	0.959	0.61	0.440	3.60	0.065
PC x FG1	1	0.20	0.658	**6.12**	**0.018**	0.50	0.483
PC x FG2	1	1.52	0.224	1.83	0.183	**6.17**	**0.019**
PC x FG3	1	1.91	0.174	0.67	0.420	0.94	0.337
FG1 x FG2	1	2.39	0.130	0.87	0.355	2.48	0.123
FG1 x FG3	1	0.32	0.576	0.83	0.366	1.19	0.281
FG2 x FG3	1	0.00	0.965	0.08	0.780	0.08	0.776
Residuals	42						
Month	11	**147.85**	**<0.001**	**154.73**	**<0.001**	**67.67**	**<0.001**
FG1 x Month	11	1.39	0.173	0.46	0.929	0.56	0.865
FG2 x Month	11	1.09	0.371	0.57	0.858	0.75	0.692
FG3 x Month	11	1.02	0.424	0.32	0.982	**3.56**	**<0.001**
PC x Month	11	1.41	0.167	0.75	0.687	1.65	0.083
PC x FG1 x Month	11	0.90	0.544	0.83	0.608	0.34	0.977
PC x FG2 x Month	11	1.25	0.250	0.44	0.939	**4.90**	**<0.001**
PC x FG3 x Month	11	1.63	0.086	0.87	0.570	0.90	0.537
FG1 x FG2 x Month	11	1.04	0.414	0.44	0.939	**2.44**	**0.006**
FG1 x FG3 x Month	11	1.36	0.188	0.86	0.583	1.60	0.095
FG2 x FG3 x Month	11	1.13	0.337	1.02	0.429	0.15	0.999
Residuals	495						

## Discussion

This study showed that removal of plant functional groups based upon traits hypothesised to affect carbon and nutrient cycling altered the response of several ecosystem processes and properties to precipitation change. Our results also provide evidence that plant functional groups have complex and interactive roles in driving function in both control and altered climate conditions. Due to our sample size, caution must be used when interpreting the results, so the results we discuss here are based upon p<0.05 rather than 0.1, a potential solution for the Type II errors that may occur in small experiments [39]. As a result, we are confident that we are reporting real effects, although some may have been overlooked. Where F values in the tables exceed 2, there is a high likelihood that a significant result would have been obtained with more replicates, e.g. the effects of FG2 upon decomposition in the second period returned F_1,42_ = 3.62, which would have been significant with higher replication, but in this case the p value was 0.064 (Table 4). Other examples of this occurred in the gas flux measures (Table 5) and extractable nutrients (Table 6). Accordingly we have underestimated the impact of functional group presence and precipitation change on grassland ecosystem function. However, we have only discussed those with p values of lower than 0.05 in this study so as to reduce the likelihood of the more serious Type I error.

### Effects of precipitation change upon ecosystem processes

The precipitation change rainfall treatment resulted in very low soil moisture levels in the summer growth period and waterlogged, possibly anoxic, conditions in the winter. This was associated with reduced rates of ecosystem *A* and R_eco_, and a similar magnitude of decrease in both processes suggests that plants are the key drivers of these responses. While *A* and R_eco_ under the precipitation change treatment showed a variety of responses depending on the functional groups present and the season, this study supports the conclusions of a meta-analysis by Wu et al. [40] which found that the net balance of *A* and R_eco_ responses to rainfall manipulations (both increased and decreased) was close to neutral, thus indicating that projected changes in rainfall patterns might not be as detrimental to soil carbon stocks as feared [31]. During the 2.5 years of this experiment, no processes were affected solely by the precipitation change treatment, thus showing that all precipitation change effects were dependent upon composition.

An unexpected result was the lagged response of soil moisture to changes in rainfall; treatment effects of the precipitation change treatment were delayed by up to six weeks, leading to stronger treatment effects on function in the spring and autumn than in the summer and winter. This highlights the importance of monitoring ecosystem function throughout the year in studies of this type. Some researchers have noticed surprisingly inconsistent relationships between ecosystem functions such as productivity [41],[42] and drought or drought alleviation, phenomena which could be explained by the lag time seen here. Studies on mixed grass prairie have demonstrated responses to a seasonal precipitation change as late as two seasons later; for example snow accumulation and melt associated with drift fences preserved ecosystem respiration levels under summer droughtby maintaining moisture in deep soil levels [27],[43].

### Effects of changes in plant functional group composition on ecosystem processes

The strong observed effects of functional group composition on ecosystem properties and process rates lends support to the view that functional group identity is instrumental in driving a range ofecosystem functions in grassland systems [44],[45]–[48]. The importance of functional group identity over species richness in driving function is supported by the finding that changes in species richness throughout the main sampling seasons of the experiment were non-significant. Despite the fact that all plots contained a perennial species from FG1, *Holcus mollis*, the dominant trend that appeared over the 2.5 years of experimentation was that process rates were higher under control conditions where a range of other perennial species (FG1) were present. This indicates that several species of this group are required to maximise function, not just a single dominant. However, when several of these species were present ecosystem processes (especially carbon flux rates) were more strongly affected by precipitation change. In contrast, carbon flux rates were much less affected by the precipitation change treatment where most of the FG1 species were absent and the other two groups were present. Morecroft and colleagues noted very similar trends in their grassland system [49]. They hypothesised that the lack of effect of summer drought on productivity in their experiment was due to gap-filling of annuals during autumn and winter, with a recovery of productivity (and consequently function) such that treatment effects were not seen in their autumn harvest. The caespitose grass and tall forb group (FG2) had consistently low abundances at our site, but the strong response of more than one ecosystem function to their removal contrasts with Grime's mass-ratio hypothesis [50], which states that species effects on ecosystem function are proportional to their biomass.

The presence of several perennial (FG1) species was a key driver of many ecosystem functions, including decomposition rates and carbon fluxes in this study. Their importance in these processes may be linked to their longevity, high LNC and deep and sparse root structures (as evidenced by their having a very similar root biomass to the much shallower rooted annuals), and the thick, dense layer of short-statured plants and associated litter they create. A more humid microclimate at the soil surface is likely to have been generated by their presence, and this combined with substantial litter inputs may have boosted microbial activity.

Decomposition responded strongly to the presence of multiple perennial species (FG1) with clear group-specific effects. Decomposition is known to be closely associated with R_eco_, and a high abundance of FG1 perennial species increased both of these processes under control conditions in the current study [51],[52]. There is also evidence that some plant species harbour species-specific microbial communities in their rhizospheres [53]. This may in turn result in greater substrate utilisation and greater R_eco_ under certain combinations of plant functional groups [54].

### Functional group identity as a regulator of ecosystem response to precipitation

Our results demonstrate that the effects of changes in rainfall on ecosystem processes can be modified by plant community composition. More specifically they show that the effects of summer drought on ecosystem processes are likely to be more substantial for communities with a high abundance of FG1 perennial species, compared to annuals, a finding which is consistent with other studies of plant community response to precipitation change [25],[55].This then has cascading effects on net photosynthetic rates and other ecosystem processes. The effect of the precipitation change treatment on soils under perennial dominated communities in the current study was smaller than that seen in some precipitation change experiments [28],[56]. This may be due to the increased winter rainfall element of our climate manipulation treatment, allowing deeper rooted species to continue to function throughout the summer drought period [14],[27].

Photosynthesis was strongly affected by a combination of the presence of multiple FG1 perennials and reducedrainfall, especially at the end of the growing season. While a reduction in process rates in response to drought is predictable, the particular response of FG1 plants is less so. When compared with the few effects of precipitation change upon the process rates of communities containing FG2 and FG3 plants, it indicates that changes in the activity, not abundance, of FG1 were responsible for the observed effects. Overall plant cover inplots containing FG1changed throughout the experiment more than in those containing the other groups, but this appeared to be a seasonal effect not a precipitation one, as shown by the lack of a three way interaction between the two treatments and time. Therefore, there is no clear link between coverand photosynthetic rate. The lack of recovery of these fluxes after drought suggests that lower soil moisture in the precipitation change treatment was associated with stomatal closure, to the detriment of photosynthetic rates in these species [57]. However, there are few studies on the effect of plant community composition on evapotranspiration in the literature, and those there are describe a positive relationship between evapotranspirationand functional group richness, possibly indicating that more diverse assemblages are less economical with water [57], though it may simply be due to higher biomass in such communities. This does not seem to be the case in our study as we found no link between evapotranspiration and the inclusion of multiple or individual functional groups. The similar magnitude of change of respiratory and photosynthetic CO_2_ flux rates over the 18 month measurement period in this study indicate that neither annual- nor perennial-dominated temperate grasslands are likely to suffer a net shift in carbon sequestration as a result of the type of rainfall changes simulated here.

The differences in gas exchange noted in this experiment were not observed for soil N levels. In control plots containing multiple FG1 species, the large N inputs from decomposing litter and increases in microbial activity, as suggested by higher R_eco_ and decomposition rates, did not appear to result in significant changes to soil extractable N. This could indicate that highly competitive perennial species balance their higher N inputs with high uptake, or possibly that their deeper, sparser root structure results in a weaker ability to prevent N leaching losses compared to annuals [58]. However, it is possible that if the experiment was better replicated or run for longer then, an effect would become apparent.

Concentrations of extractable soil P were low but significantly affected by precipitation, functional group identity and the interaction between these throughout the year. This grassland is co-limited by N and P [59] and our results indicate that it is the P cycle that is more sensitive to changes in precipitation and plant community composition. In general where several perennial (FG1) species were present P availability was higher. This may be due to the low abundance of legumes in this group, which have a high P demand and are likely to reduce soil P concentrations more than other species [60]–[62]. Additionally, there is some evidence that deeper rooted species are able to increase the net labile P pool by taking up P from deeper soil layers, which could account for the more P-rich soils under perennial-dominated communities, and the overall increase in soil P in the soils as the experiment continued [63].

In general P was lower when caespitose grasses (FG2) were present, and the precipitation change treatment only altered P when this group was present. This suggests that the mechanisms of P uptake and availability in plots containing FG2 were strongly affected by soil moisture levels or poor ability to prevent leaching. Species in this group have a low foliar N∶P ratio but a high biomass and they are notable for their high nutrient uptakes and fast growth rates, particularly *Dactylis glomerata* and *Lolium perenne* [64],[65]. They are also known for their resilience to drought [66]. However, our findings suggest that the presence of this group could result in very P-limited rhizospheres, particularly during the spring when the germination and establishment of annuals occurs, and this could impede colonisation by other species.

As there were relatively few significant, and/or synergistic interactions between the presence of functional groups there was little support for ageneral positive relationship between ecosystem process rates and the number of functional groups present, which is unexpected given the vast literature on the subject [11],[60],[67]. The clearest relationships have traditionally been with productivity, which we did not measure directly in this study (see [68] for a comprehensive review). The disparity of these findings may be due to a number of factors. These include the use of trait-defined functional groups as opposed to arbitrary groupings such as the commonly used grass/legume/herb classification [22],[44],[67]. Categorising species into non-arbitrary functional groups defined by traits that are likely to influence the measured functions may increase the relative importance of functional group identity effects and reduce the strength of the diversity *per se* effect. A second reason may be due to methodology; instead of constructing communities from random sets of species, as is the norm in biodiversity-ecosystem function studies [11],[15], we removed species from natural communities while leaving the dominant species present in all plots. Such an approach, which better reflects real extinction scenarios, has been seen to result in a weaker relationship between diversity and function than is seen in artificially assembled communities [69].

### Semi-natural grasslands in a changing world

From our results it can be inferred that, while the perennial plants of FG1 are important drivers of ecosystem function and theirrelationship with it is sensitive to climate change, annual plants may help maintain function during periods of water stress. This is particularly apparent for ecosystem C fluxes, which are substantially reduced when water is limiting [39]. There is some evidence, both from the current study and more generally, to suggest that traits characteristic of perennial species may make them susceptible to future drought [25]. From our results, it seems possible that while cover of these species would not change, function would be reduced. In particular, the allocation of roots to deeper soil layers could have prevented them from optimising water capture in the rainfall scenario in this experiment, thus explaining their reduced process rates under altered rainfall scenarios. An associated competitive release of annual forbs may also increase species diversity under future climate scenarios [14]. This seems to contradict the widespread support for the idea that deeper rooted species are more drought resistant, most commonly demonstrated in arid or semi- arid landscapes [70],[71]. However, we hypothesise that in our precipitation change treatment the contrast of very small rainfall pulses with sporadic high rainfall favoured the morphology of shallow rooted species which could utilise small volumes before they were lost to evaporation [6]. The lack of observed precipitation change effects on process rates in systems containing annuals could, therefore, indicate that temperate grassland systems containing this group may be more resistant to future climate change than previously thought. It should be noted, however, that the results presented here are from only 2.5 years of modest, though realistic, reductions in summer rainfall; more extreme changes in rainfall patterns, over longer timescales, or extreme weather events could have much greater ecological consequences.

Many global change drivers are known to affect grassland community composition, and large changes can be expected throughout the coming century [2],[31]. Perennial species generally dominate grasslands in temperate Europe, but these systems may be more vulnerable to changes in water inputs than systems dominated by annual species and/or caespitose grasses, which offer different life histories and strategies to cope with changing patterns of water availability. By grouping species in terms of trait complexes, differing responses to future changes in precipitation patterns can be shown in terms of gas fluxes and nutrient cycles. Our results indicate that future grassland management should aim to accommodate both perennial and annual species. The latter are often in low abundance in the improved (fertilised and sown) grasslands that are common in Europe [72], but may help to maintain ecosystem function and the associated delivery of ecosystem services in future climates.

## Supporting Information

Text S1 **Supplementary methods.**
(DOCX)

Figure S1 **Map, plot schematic and preliminary site characterisation of the DIRECT field site.** Image of the field site of the DIRECT experiment in June 2009, accessed from Google maps 11/11/10, co-ordinates 51.4091°N, 0.6378°W. The dark blue and red rectangles delimit ongoing experiments in 2006 and 2007. The road runs parallel to the 105.8 m long periphery of the field, ∼20 m away. The shelters slope into the prevailing wind. Block 1 is surrounded by light blue, through to block 4 in green. Plots without roofs belong to a related experiment.
(DOCX)

Figure S2 **Schematic of the dimensions of the rainout shelters.**
(DOCX)

Figure S3 **Vegetation cover by functional diversity treatment throughout the experiment.** Weeding was carried out in August 2008, May 2009 and June 2010.
(DOCX)

Figure S4 **Effect of treatments upon** ***H. mollis*** **throughout the experiment.** The FG in the legend refers to presence of these functional groups in the plots.
(DOCX)

Table S1 **The list of the plant species in the field site in their allocated functional groups.**
(DOCX)

Table S2 **Percentage cover estimates of plots containing each functional group in turn at the beginning and end of the experiment.** Due to overlap of species the values may exceed 100%.
(DOCX)

Table S3 **Linear mixed effects model evaluating effect of treatments on** ***H. mollis*** **coverage over time.** FGx refers to the presence of the functional group in question, PC to the precipitation change treatment.
(DOCX)
