# Supplementary material for: Plant Functional Group Composition Modifies the Effects of Precipitation Change on Grassland Ecosystem Function
Source: PLoS One. 2013 Feb 20;8(2):e57027. doi: 10.1371/journal.pone.0057027 (PMC3577764; doi:10.1371/journal.pone.0057027)
Supplement: Text S1 — Supplementary methods. (DOCX) [file pone.0057027.s001.docx]

**Text S1** Supplementary methods

Four soil samples from each plot were collected in October 2008 for analysis (n=56). The plot samples were homogenised and sieved, and 1:1 wet soil to dH_2_O was mixed. The pH was measured with a pH meter (Mettler Toledo, Leicester, UK). They were then analysed using a one-way ANOVA with block as a factor, and then a post-hoc Tukey’s HSD test to find that the first block (at the lowest point of the incline) had a significantly higher pH than the other three blocks (Block 1 mean = 5.87±0.05, Combined blocks 2-4 mean = 5.59±0.04, F_3,51_=5.15, p<0.01).

A soil augur was used (10.5cm depth, 3cm diameter, 23.63cm^3^ volume) to collect two soil samples from every plot. These samples were dried overnight at 60˚C before weighing. The bulk density was calculated using the equation weight/volume, and analysed using a one-way ANOVA as above, which found that there is no difference in compaction or pore space across the site (F_3,52_= 2.36, p>0.05).

Four samples of soil from each plot were homogenised and dried, before being passed through sieves that had aperture sizes approximate to USDA particle size classifications (0.002μm, 0.063μm- proxy for 0.05μm, 0.09μm- proxy for 0.1μm, 0.425μm instead of 0.5μm, 1mm and 2mm, USDA 2010a). The USDA soil triangle was used to describe the site’s soil as loamy sand (93% sand, various grades, 7% silt). Each soil grade was arcsine transformed and blocks compared in an ANOVA with a post-hoc Tukey’s HSD test. None of the soil grades changed across the site, except medium sized sand, which occurred significantly less (34.78%±2.93) in block 1 than in block 4 on the top of the incline (52.06%±3.21, F_3,10_=3.46, p<0.01).

Two soil pits were dug, one at either end of the site, to ascertain the rooting and soil depth. The rooting and soil depths were shallower at the south-eastern side (20cm rooting depth, 39cm soil depth in block 1, 35cm rooting depth, 51cm soil depth at block 4).

In order to recreate the summer rainfall projection, individual rainout shelters were designed, which were intended to be durable, easily removed and replaced and to allow as much sunlight transmission as possible. The design of prototypes, showed transparent corrugated Corolux PVC was cost effective, easily replaced and very durable, mounted upon a square frame and at an angle of 12˚, enough to allow the water to run off. Light transmission was on average 68.76% of ambient light. The frames were angled into the prevailing wind (lowest end SW) and 0.9m high at the front, 1.4m high at the back. These shelters were put up between June and August each year, and over both ambient and climate change treatments to control for differences in microclimatic and light conditions between treatments, as well as seed dispersal. The design is mainly based upon fixed-location shelters in Argentina created by Yahdjian and Sala [28]. The ambient plots also had plastic sheeting, but many holes were drilled in the grooves to allow all water to fall through.
