# Supplementary material for: Plant Functional Group Composition Modifies the Effects of Precipitation Change on Grassland Ecosystem Function
Source: PLoS One. 2013 Feb 20;8(2):e57027. doi: 10.1371/journal.pone.0057027 (PMC3577764; doi:10.1371/journal.pone.0057027)

**Figure S1:** Map, plot schematic and preliminary site characterisation of the DIRECT field site.

Image of the field site of the DIRECT experiment in June 2009, accessed from Google maps 11/11/10, co-ordinates 51.4091ºN, 0.6378ºW. The dark blue and red rectangles delimit ongoing experiments in 2006 and 2007. The road runs parallel to the 105.8m long periphery of the field, ~20m away. The shelters slope into the prevailing wind. Block 1 is surrounded by light blue, through to block 4 in green. Plots without roofs belong to a related experiment.


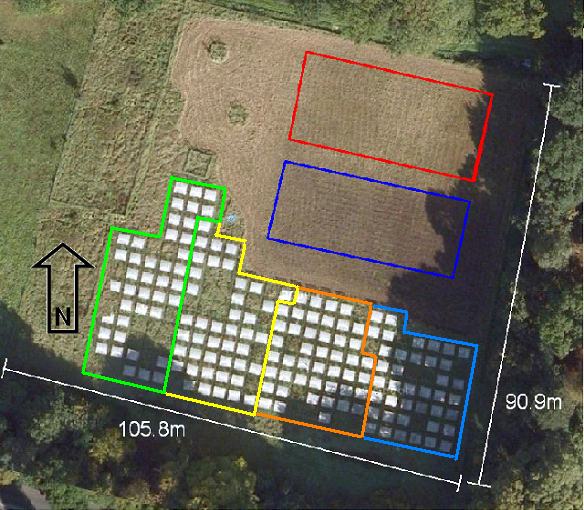

Supplement: Figure S1 — Map, plot schematic and preliminary site characterisation of the DIRECT field site. Image of the field site of the DIRECT experiment in June 2009, accessed from Google maps 11/11/10, co-ordinates 51.4091°N, 0.6378°W. The dark blue and red rectangles delimit ongoing experiments in 2006 and 2007. The road runs parallel to the 105.8 m long periphery of the field, ∼20 m away. The shelters slope into the prevailing wind. Block 1 is surrounded by light blue, through to block 4 in green. Plots without roofs belong to a related experiment. (DOCX) [file pone.0057027.s002.docx]
