# Supplementary figures and images for: Plant Functional Group Composition Modifies the Effects of Precipitation Change on Grassland Ecosystem Function
Source: PLoS One. 2013 Feb 20;8(2):e57027. doi: 10.1371/journal.pone.0057027 (PMC3577764; doi:10.1371/journal.pone.0057027)

**Figure S2** Schematic of the dimensions of the rainout shelters.


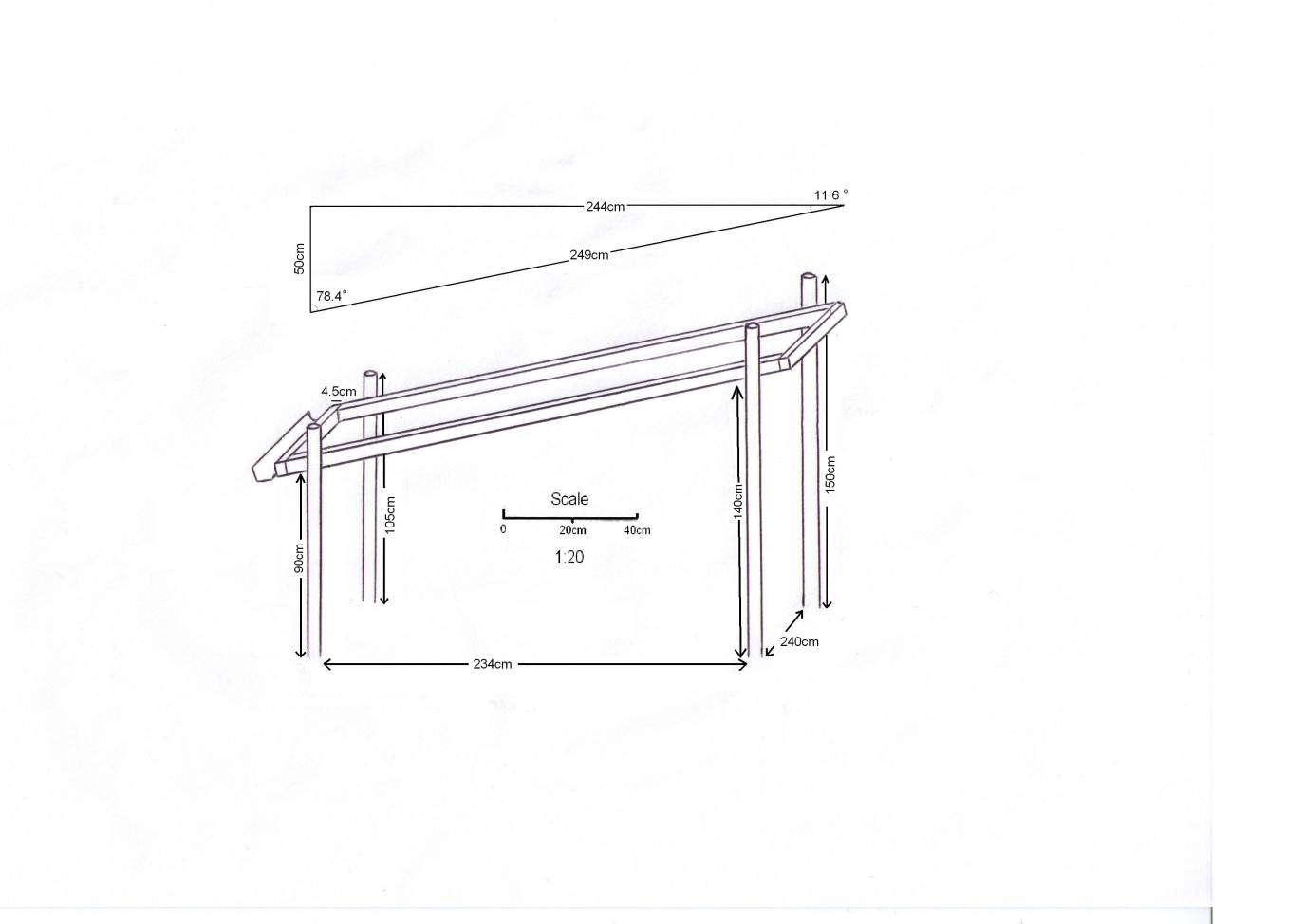

Supplement: Figure S2 — Schematic of the dimensions of the rainout shelters. (DOCX) [file pone.0057027.s003.docx]
