# Supplementary material for: Plant Functional Group Composition Modifies the Effects of Precipitation Change on Grassland Ecosystem Function
Source: PLoS One. 2013 Feb 20;8(2):e57027. doi: 10.1371/journal.pone.0057027 (PMC3577764; doi:10.1371/journal.pone.0057027)

**Figure S3** Vegetation cover by functional diversity treatment throughout the experiment. Weeding was carried out in August 2008, May 2009 and June 2010.


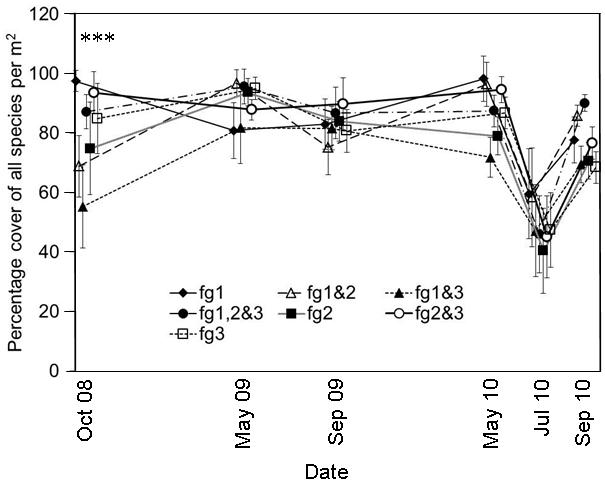

Supplement: Figure S3 — Vegetation cover by functional diversity treatment throughout the experiment. Weeding was carried out in August 2008, May 2009 and June 2010. (DOCX) [file pone.0057027.s004.docx]
