# Supplementary material for: Plant Functional Group Composition Modifies the Effects of Precipitation Change on Grassland Ecosystem Function
Source: PLoS One. 2013 Feb 20;8(2):e57027. doi: 10.1371/journal.pone.0057027 (PMC3577764; doi:10.1371/journal.pone.0057027)

**Figure S4** Effect of treatments upon *H. mollis* throughout the experiment. The FG in the legend refers to presence of these functional groups in the plots.


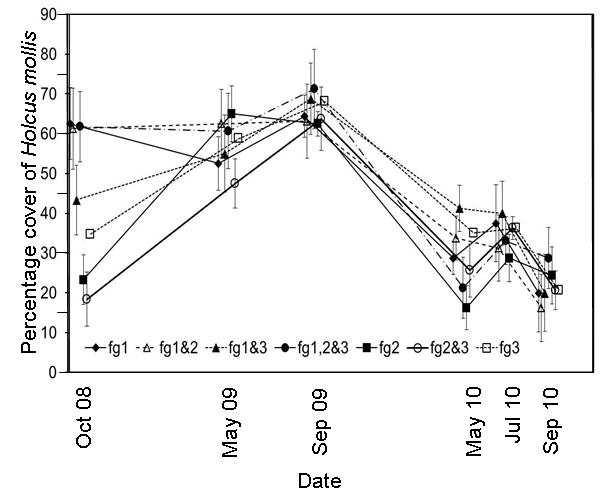

Supplement: Figure S4 — Effect of treatments upon H. mollis throughout the experiment. The FG in the legend refers to presence of these functional groups in the plots. (DOCX) [file pone.0057027.s005.docx]
