# Supplementary material for: Plant Functional Group Composition Modifies the Effects of Precipitation Change on Grassland Ecosystem Function
Source: PLoS One. 2013 Feb 20;8(2):e57027. doi: 10.1371/journal.pone.0057027 (PMC3577764; doi:10.1371/journal.pone.0057027)
Supplement: Table S1 — The list of the plant species in the field site in their allocated functional groups. (DOCX) [file pone.0057027.s006.docx]

**Table S1** The list of the plant species in the field site in their allocated functional groups.

| **Functional group 1**  ‘Perennials’ | **Functional group 2**  ‘Caespitose grasses’ | **Functional groups 3**  ‘Annuals’ |
| --- | --- | --- |
| *Achillea millefolium* | *Agrostis stolonifera* | *Apera spica-venti* |
| *Agrostis capillaris* | *Arrhenatherum elatius* | *Atriplex patula* |
| *Agrostis gigantea* | *Avena fatua* | *Bromus hordeaceus* |
| *Anthoxanthum odoratum* | *Dactylis glomerata* | *Bromus sterilis* |
| *Bellis perennis* | *Galium aparine* | *Capsella bursa-pastoris* |
| *Cirsium arvense* | *Lapsana communis* | *Cerastium fontanum* |
| *Cynosurus cristatus* | *Lolium perenne* | *Cerastium glomeratum* |
| *Dipsacus fullonum* | *Vulpia bromoides* | *Chenopodium album* |
| *Elytrigia repens* |  | *Crepis capillaris* |
| *Festuca rubra* |  | *Geranium molle* |
| *Glechoma hordeacea* |  | *Matricaria recutita* |
| *Holcus lanatus* |  | *Medicago lupulina* |
| *Lotus corniculatus* |  | *Myosotis arvensis* |
| *Plantago major* |  | *Plantago lanceolata* |
| *Poa trivialis* |  | *Poa annua* |
| *Prunella vulgaris* |  | *Raphanus raphanistrum* |
| *Ranunculus repens* |  | *Senecio jacobaea* |
| *Rumex acetosa* |  | *Senecio vulgaris* |
| *Rumex acetosella* |  | *Sisymbrium officinale* |
| *Rumex obtusifolius* |  | *Sonchus asper* |
| *Stellaria graminea* |  | *Stellaria media* |
| *Taraxacum officinale* |  | *Trifolium dubium* |
| *Trifolium repens* |  | *Trifolium pratense* |
| *Urtica dioica* |  | *Tripleurospermum inodorum* |
|  |  | *Veronica arvensis* |
|  |  | *Vicia hirsute* |
|  |  | *Vicia sativa* |
|  |  | *Vicia tetrasperma* |
