# Supplementary material for: Plant Functional Group Composition Modifies the Effects of Precipitation Change on Grassland Ecosystem Function
Source: PLoS One. 2013 Feb 20;8(2):e57027. doi: 10.1371/journal.pone.0057027 (PMC3577764; doi:10.1371/journal.pone.0057027)
Supplement: Table S2 — Percentage cover estimates of plots containing each functional group in turn at the beginning and end of the experiment. Due to overlap of species the values may exceed 100%. (DOCX) [file pone.0057027.s007.docx]

**Table S2** Percentage cover estimates of plots containing each functional group in turn at the beginning and end of the experiment. Due to overlap of species the values may exceed 100%.

| Month | FG1 present | FG1 absent | FG2 present | FG2 absent | FG3 present | FG3 absent |
| --- | --- | --- | --- | --- | --- | --- |
| October 2008 | 101.7% | 54.9% | 75.9% | 89.2% | 78.2% | 86.2% |
| September 2010 | 76.3% | 69.2% | 80.8% | 71.9% | 76.1% | 78.1% |
