# Supplementary material for: Plant Functional Group Composition Modifies the Effects of Precipitation Change on Grassland Ecosystem Function
Source: PLoS One. 2013 Feb 20;8(2):e57027. doi: 10.1371/journal.pone.0057027 (PMC3577764; doi:10.1371/journal.pone.0057027)
Supplement: Table S3 — Linear mixed effects model evaluating effect of treatments on H. mollis coverage over time. FGx refers to the presence of the functional group in question, PC to the precipitation change treatment. (DOCX) [file pone.0057027.s008.docx]

**Table S3** Linear mixed effects model evaluating effect of treatments on *H. mollis* coverage over time. FGx refers to the presence of the functional group in question, PC to the precipitation change treatment.

| Treatment | |  | *Holcus mollis* cover | |
| --- | --- | --- | --- | --- |
|  | d.f | | F | P |
| Intercept | **1** | | **937.19** | **<0.001** |
| PC | 1 | | 2.77 | 0.103 |
| FG1 present | 1 | | **5.89** | **0.020** |
| FG2 present | 1 | | 0.52 | 0.474 |
| FG3 present | 1 | | **0.49** | 0.487 |
| PC x FG1 | **1** | | 2.59 | 0.115 |
| PC x FG2 | 1 | | 0.16 | 0.691 |
| PC x FG3 | 1 | | **0.11** | 0.746 |
| FG1 x FG2 | 1 | | 2.35 | 0.133 |
| FG1 x FG3 | 1 | | 0.04 | 0.836 |
| FG2 x FG3 | 1 | | 0.06 | 0.809 |
| Residuals | 42 | |  |  |
| Month | 5 | | **35.15** | **<0.001** |
| FG1 x Month | 5 | | **4.78** | **<0.001** |
| FG2 x Month | 5 | | 1.34 | 0.250 |
| FG3 x Month | 5 | | 0.64 | 0.672 |
| PC x Month | 5 | | 0.12 | 0.987 |
| PC x FG1 x Month | 5 | | 0.78 | 0.564 |
| PC x FG2 x Month | 5 | | 0.45 | 0.816 |
| PC x FG3 x Month | 5 | | 0.65 | 0.662 |
| FG1 x FG2 x Month | 5 | | 0.77 | 0.571 |
| FG1 x FG3 x Month | 5 | | 0.99 | 0.424 |
| FG2 x FG3 x Month | 5 | | 1.32 | 0.255 |
| Residuals | 225 | |  |  |
